# Evaluation of the Phosphoproteome of Mouse Alpha 4/Beta 2-Containing Nicotinic Acetylcholine Receptors In Vitro and In Vivo

**DOI:** 10.3390/proteomes6040042

**Published:** 2018-10-15

**Authors:** Megan B. Miller, Rashaun S. Wilson, TuKiet T. Lam, Angus C. Nairn, Marina R. Picciotto

**Affiliations:** 1 Department of Psychiatry, Yale University School of Medicine, 34 Park Street, 3rd Floor Research, New Haven, CT 06508, USA; miller.meganb@gmail.com (M.B.M.); Angus.Nairn@yale.edu (A.C.N.); 2 Yale/NIDA Neuroproteomics Center, 300 George Street, New Haven, CT 06509, USA; rashaun.wilson@yale.edu (R.S.W.); TuKiet.Lam@yale.edu (T.T.L.); 3 W.M. Keck Biotechnology Resource Laboratory, Yale University School of Medicine, 300 George Street, New Haven, CT 06509, USA; 4 Department of Molecular Biophysics and Biochemistry, Yale University, 266 Whitney Avenue, New Haven, CT 06520, USA

**Keywords:** nicotinic receptor, CaMKII, PKA, quantitative phosphoproteomics, mouse, phosphorylation, nicotine

## Abstract

Activation of nicotinic acetylcholine receptors containing α4 and β2 subunits (α4/β2* nAChRs) in the mammalian brain is necessary for nicotine reinforcement and addiction. We previously identified interactions between α4/β2* nAChRs and calcium/calmodulin-dependent protein kinase II (CaMKII) in mouse and human brain tissue. Following co-expression of α4/β2 nAChR subunits with CaMKII in HEK cells, mass spectrometry identified 8 phosphorylation sites in the α4 subunit. One of these sites and an additional site were identified when isolated α4/β2* nAChRs were dephosphorylated and subsequently incubated with CaMKII in vitro, while 3 phosphorylation sites were identified following incubation with protein kinase A (PKA) in vitro. We then isolated native α4/β2* nAChRs from mouse brain following acute or chronic exposure to nicotine. Two CaMKII sites identified in HEK cells were phosphorylated, and 1 PKA site was dephosphorylated following acute nicotine administration in vivo, whereas phosphorylation of the PKA site was increased back to baseline levels following repeated nicotine exposure. Significant changes in β2 nAChR subunit phosphorylation were not observed under these conditions, but 2 novel sites were identified on this subunit, 1 in HEK cells and 1 in vitro. These experiments identified putative CaMKII and PKA sites on α4/β2* nAChRs and novel nicotine-induced phosphorylation sites in mouse brain that can be explored for their consequences on receptor function.

## 1. Introduction

High-affinity nicotinic acetylcholine receptors containing the α4 and β2 subunits (α4/β2* nAChRs, where * denotes other, potentially unidentified, subunits) are essential for the rewarding and reinforcing properties of nicotine in the mouse [1,2,3,4]. α4/β2* nAChRs are intrinsic ion channel-containing proteins that flux positive ions, including calcium, in response to nicotine or the endogenous neurotransmitter acetylcholine. Activation of α4/β2* nAChRs depolarizes neurons on which they are expressed, leading to changes in intracellular signaling, such as activation of calcium-dependent kinases [5].

In addition to initiating calcium signaling, nicotine also increases the number of nAChRs and can alter the associated proteome of α4/β2* nAChRs in mouse and human brain [6]. Biochemical studies have identified a number of interacting proteins that regulate assembly, trafficking, and function of α4/β2* nAChRs. For example, the chaperone 14-3-3 has been identified as an α4 nAChR subunit interactor in multiple studies [6,7,8], and this interaction can alter the physiological properties of α4/β2* nAChRs [7,9,10]. Interestingly, the association between 14-3-3 and the α4 nAChR subunit is regulated by protein kinase A (PKA), and is critical for regulating the desensitization kinetics of the receptor [7,10,11,12]. Other kinases can also regulate nAChR function. For example, phosphorylation of the α4 nAChR subunit by the calcium-dependent protein kinase PKC, as well as dephosphorylation by the calcium-dependent phosphatase calcineurin, also regulate desensitization of α4/β2* nAChRs in response to prolonged nicotine exposure [13,14,15]. Thus, biochemical studies have established an important role for nAChR phosphorylation in regulation of nicotine signaling through its receptors.

Several studies have now evaluated the α4/β2* nAChR-associated proteome, and these studies have identified several proteins that co-immunoprecipitate with the receptor from mouse and human brain [6,8,16]. Interestingly, a quantitative interaction between α4/β2* nAChRs and multiple isoforms of calcium/calmodulin-dependent protein kinase II (CaMKII) has been identified in mouse and human brain tissue [6,8]. This is of particular interest because activation of nAChRs by nicotine could activate associated CaMKII directly, leading to phosphorylation of nAChR subunits or of downstream targets. In mice, acute nicotine exposure activates CaMKII in the spinal cord [17] and brain [18], whereas chronic exposure increases CaMKII activity in the nucleus accumbens [19], all of which require α4/β2* nAChRs. CaMKII is also required for development of anxiety-like behaviors during nicotine withdrawal [20]. Taken together, these studies suggest that nAChR-mediated activation of CaMKII is important for at least a subset of the behavioral effects of nicotine related to addiction, and the direct interaction between α4/β2* nAChRs and CaMKII isoforms provides the rationale for determining whether nAChR subunits are substrates for phosphorylation.

In the current set of experiments, we used mass spectrometry to identify the residues phosphorylated on the α4 and β2 nAChR subunits when co-expressed with CaMKIIα in HEK cells, when dephosphorylated and subjected to phosphorylation in vitro with CaMKIIα or PKA, and when isolated from mouse brain at baseline, or following exposure to acute or repeated nicotine in vivo. These studies were designed to determine whether CaMKII can phosphorylate the α4 and β2 nAChR subunits in cells that do not normally express these proteins, and whether sites identified in the cellular assay were recapitulated when purified nAChRs were incubated with CaMKIIα or a kinase that is endogenously expressed in HEK cells (PKA) in vitro. The in vitro study also allowed us to determine whether previously identified PKA sites [7,10,11,12] could be identified in our studies. Finally, we provide the first evidence of nAChR phosphorylation in mouse brain, at baseline and following nicotine exposure in vivo.

## 2. Materials and Methods

### 2.1. Animals

Adult male C3H mice (approval number: 2016-07895) were obtained from Jackson laboratories and housed in groups of no more than 5 individuals per cage, maintained on a 12:12 h light/dark cycle, and given ad libitum access to food and water. All procedures involving animals were approved by the Yale University Institutional Animal Care and Use Committee and conformed to the standards for animal care and use set by the National Institutes of Health.

### 2.2. Cell Culture

HEK-293 (HEK) cells (ATCC) were grown and maintained in a humidified incubator at 37 °C and 5% CO_2_. DMEM cell culture medium supplemented with 10% fetal bovine serum (FBS, Gibco) and antibiotic/antimycotic (Gibco), according to established ATCC protocols. Prior to transient transfection, HEK cells were split and plated at medium-high density on plastic 10 cm dishes which were pre-treated with 0.05 mg/mL poly-d-lysine (PDL) in water.

### 2.3. Cell Transfection

Transient expression of nAChR subunits, mRuby, and CaMKII-mRuby were performed in serum-free medium (SFM) using Lipofectamine 2000 (Invitrogen, Carlsbad, CA, USA) at 2.5 μL per μg DNA. For cell-based phosphorylation assays, the following combinations of plasmids were used, each in triplicate: (1) untagged α4-nAChR + β2-nAChR-YFP + mRuby, (2) untagged α4-nAChR + β2-nAChR-YFP + CaMKII-mRuby, (3) α4-nAChR-YFP + Untagged β2-nAChR + mRuby, and (4) α4-nAChR-YFP + Untagged β2-nAChR + CaMKII. For in vitro phosphorylation assays, HEK cells were transfected with either untagged α4-nAChR + β2-nAChR-YFP or with α4-nAChR-YFP + Untagged β2-nAChR. For all experiments, transfection suspensions were prepared by combining DNA and Lipofectamine in a small volume of SFM and incubating at room temperature for 30 min. Transfection suspensions were added to cells with an additional volume of SFM, and returned to the incubator for 24 h prior to harvesting.

### 2.4. Plasmids

All nAChR plasmids were generous gifts from Henry Lester [21], and can be procured from Addgene (Cambridge, MA, USA): nAChR alpha4 WT (Addgene plasmid #24271), nAChR alpha4-YFP (Addgene plasmid #15245), nAChR beta2 WT (Addgene plasmid #24272), and nAChR Beta2-YFP (Addgene plasmid #15107). CaMKIIα-mRuby2 was created from full length Camui-CR (a gift from Michael Lin; Addgene plasmid #40256; [22]) using the *Nhe*1/*Apa*1 cloning site. mRuby2-C1 was purchased from Addgene (plasmid #54768; [22]).

### 2.5. HEK Cell Harvest and Protein Extraction

24 h after transfection, HEK transients were harvested by scraping cells into ice-cold membrane extraction buffer (MEB; 50 mM Tris-HCl (pH 7), 120 mM NaCl, 5 mM KCl, 1 mM MgCl_2_) containing 2% Triton X-100, protease inhibitors (PMSF and Sigma protease inhibitor cocktail), and phosphatase inhibitors (5 mM sodium fluoride, 0.1 mM sodium orthovanadate, and Sigma phosphatase inhibitor cocktails 2 and 3). For in vitro phosphorylation assays, only the baseline samples were collected in the presence of phosphatase inhibitors; separate samples for the lambda phosphatase, PKA, and CaMKIIα conditions were collected in ice-cold MEB without phosphatase inhibitors. Harvested cells underwent 2 rounds of sonication/vortex cycles on ice, and were then allowed to incubate on ice for ~30 min to facilitate solubilization. Insoluble material was removed by brief centrifugation, and the resulting supernatants were used for further experiments.

### 2.6. In Vivo Nicotine Treatment

The nicotine treatment paradigm used was essentially as we have used previously [23,24]. Adult C3H mice were randomly assigned to each of three groups containing 5 animals each: Control, Acute, and Chronic. Animals were given ad libitum access to food and water containing either 200 μg/mL nicotine hydrogen tartrate (calculated as free base) in 2% (*w*/*v*) saccharin (chronic condition only) or 2% saccharin with molar-matched tartaric acid (Sigma-Aldrich, St. Louis, MO, USA; Acute and Control conditions) for 14 days. Animals were housed 3–4 to a cage, and pairings were set up at least 5 days prior to introduction of experimental water to allow for acclimation. Water was stored in darkened bottles to protect from light.

At the end of two weeks, animals were treated with a single, subcutaneous dose of either nicotine (0.5 mg/kg, acute condition), or saline (chronic and control conditions). Experimental drinking water was removed from cages 1 h prior to dosing, and animals were sacrificed by cervical dislocation 15 min after dosing. Whole brains were immediately removed on ice, then flash-frozen and stored at −80 °C until processing.

### 2.7. Brain Tissue Processing for Immunoprecipitation

On the day of tissue preparation, frozen brains were thawed on ice and homogenized in 10 volumes of ice-cold tissue homogenization buffer (10 mM HEPES [pH 7.4], 320 mM sucrose, 2 mM EDTA) containing protease inhibitors (PMSF and Sigma protease inhibitor cocktail) and a panel of phosphatase inhibitors (5 mM sodium fluoride, 0.1 mM sodium orthovanadate, and Sigma phosphatase inhibitor cocktails 2 and 3). Lysates were subjected to two rounds of sonication and vortexing, incubated on ice for 30 min, then spun at 1000× *g* for 10 min. Pellets were discarded, and “S1” supernatants were transferred to clean ultracentrifuge tubes and spun for 1 h at 100,000× *g* and 4 °C using a Beckman 70.1 Ti rotor. The resulting supernatants were removed and the pellets (“P2”) were resuspended in 0.3× their initial volume in membrane extraction buffer (MEB; 50 mM Tris-HCl (pH 7), 120 mM NaCl, 5 mM KCl, 1 mM MgCl_2_) containing 2% Triton X-100, protease inhibitors (PMSF and Sigma protease inhibitor cocktail), and phosphatase inhibitors (5 mM sodium fluoride, 0.1 mM sodium orthovanadate, and Sigma phosphatase inhibitor cocktails 2 and 3). Samples were vortexed thoroughly, and allowed to incubate on ice for ~2 h to facilitate solubilization. P2 homogenates were transferred to microfuge tubes and spun at 1000× *g* and 4 °C in for 10 min. The supernatant (S3; Triton-soluble crude membrane fraction) was used for immunoprecipitation of nAChRs.

### 2.8. Immunoprecipitation

Immunoprecipitation (IP) of HEK transients was conducted using a magnetic GFP-nAb resin from Allele, essentially according to manufacturer protocols. Briefly, GFP-nAb resin was washed 3× with binding buffer (10 mM Tris-HCl, 150 mM NaCl, pH 7.5), then resuspended and aliquot into microfuge tubes (20 μL resin/IP). While preparing resin, HEK cell lysates were thawed on ice and diluted in binding buffer such that the detergent concentration was not higher than 0.6% (*v*/*v*). Wash buffer was completely removed from resin using a magnetic stand, and diluted HEK lysates were added to washed resin. IP was conducted by tumbling overnight at 4 °C.

The following day, bound material was separated from the unbound supernatant by placing resin on a magnetic stand for at least 2 min. Supernatants were removed and resin was washed 1× in binding buffer and 2× in wash buffer (high salt Tris Buffered Saline). Bound fractions of HEK lysates from co-expression experiments (co-expression of nAChRs with CaMKII or mRuby in HEKs) and baseline samples from in vitro phosphorylation experiments were eluted by boiling resin in in 60 μL 1× Laemmli sample buffer (Bio-Rad, Hercules, CA, USA) with SDS (prepared in ultrapure water for MS). Bound fractions of HEK lysates, intended for in vitro phosphorylation experiments, were treated according to the dephosphorylation/phosphorylation protocol(s) detailed below, prior to eluting.

Immunoprecipitation of α4/β2-containing nAChRs from mouse brain lysates was done using M-270 Epoxy magnetic Dynabeads (Invitrogen, Carlsbad, CA, USA) linked to purified nAChR antiserum. Approximately 600 μg of rat-anti-α4 (mAb299; Lindstrom) was combined with 175 μg of rat-anti-beta2 (mAb270; Lindstrom) and linked to surface-activated M-270 Epoxy Dynabeads (Invitrogen; 5 mg Dynabeads /100 μg mAb) according to the manufacturer’s instructions. mAb299 and mAb270 antisera were a generous gift from Jon Lindstrom, and were characterized previously [25]. Antibody linking was conducted overnight (~23 h) at room temperature with gentle agitation. The following day, linked resin was separated on a magnetic stand, rinsed with Phosphate Buffered Saline (PBS) + 0.1% BSA (*w*/*v*), then resuspended in PBS and stored at 4 °C until use. On the day of IP, linked resin was rinsed once in PBS and then equally divided into each of 15 clearly labeled microfuge tubes (5 samples × 3 treatment groups). Processed whole brain samples (S3 fraction) were added to the prepared resin, and IP was conducted by tumbling overnight at 4 °C. The next day, beads and bound proteins were separated on a magnetic stand and rinsed 3× with PBS and 1× with PBS containing 0.1% BSA (*w*/*v*) and 0.1% Triton X-100 (*v*/*v*). The final wash was then removed, and the bound fractions were eluted by boiling resin in 50 μL 1× Laemmli sample buffer (Bio-Rad) with SDS. Once cooled, the eluate was immediately removed from the resin and stored at −20 °C until gels were run.

### 2.9. In Vitro Phosphorylation

Transfected HEK cells for in vitro phosphorylation experiments were separated into four groups: baseline, lambda phosphatase, PKA, and CaMKII. Baseline nAChR transients were collected in the presence of phosphatase inhibitors, and eluted immediately following IP, as described above. The remaining three groups were harvested in the absence of phosphatase inhibitors, and immunoprecipitated receptors were subject to in vitro dephosphorylation with purified lambda phosphatase (New England Biolabs, Ipswich, MA, USA; all but baseline group received this treatment).

#### 2.9.1. Dephosphorylation with Lambda Phosphatase

Immunoprecipitated receptors were dephosphorylated in vitro using recombinant lambda phosphatase (New England Biolabs), according to manufacturer instructions. Briefly, immunoprecipitated receptors (still bound to nAb-GFP resin) were rinsed once with 200 μL PMP phosphatase buffer (manufacturer supplied) to equilibrate. The following phosphatase mixture was then added to each tube: 100 μL PMP buffer, 10 μL MnCl_2_ (manufacturer supplied), 1 μL each of aprotinin and PMSF, and 2 μL of lambda phosphatase. Dephosphorylation continued for 1 h at 30 °C with gentle agitation. After incubation, resins were separated from dephosphorylation mixture by placing on a magnetic stand, washed with PMP, and dephosphorylation was conducted a second time. Following the second treatment, 50 μL of 500 mM EDTA was added to each sample and suspensions incubated on ice for 5 min to stop phosphatase activity. Supernatants were then removed, and resin was washed 2× in wash buffer. Bound receptors in the lambda phosphatase group were eluted here in 40 μL 1× Laemli Sample Buffer (LSB) prepared in ultrapure water. Remaining samples continued on to rephosphorylation with PKA or CaMKIIα.

#### 2.9.2. Phosphorylation with PKA

Following dephosphorylation, samples in the PKA group were phosphorylated with purified PKA (New England Biolabs) according to the manufacturer’s instructions. Briefly, bound receptors were rinsed in 1× PK buffer (manufacturer supplied), and then incubated in the following PKA phosphorylation mixture: 100 μL 1× PK buffer, 1 μL of sigma protease inhibitor cocktail, 2 μL of 10 mM ATP stock (200 μM total), and 2 μL of PKA enzyme. Phosphorylation continued for 1–2 h at 30 °C with gentle agitation. Following incubation, supernatants were removed, and resin was washed 2× in wash buffer. Bound receptors were then eluted in 40 μL 1× LSB prepared in ultrapure water.

#### 2.9.3. Phosphorylation with CaMKIIα

Following dephosphorylation, samples in the CaMKIIα group were phosphorylated with purified CaMKIIα (New England Biolabs) according to manufacturer instructions. CaMKIIα enzyme was activated by combining 2 μL of the purified kinase with 100 μL buffer, 200 μM ATP, 1.2 μL calmodulin, and 2 mM CaCl_2_, all provided by the manufacturer. The activation solution incubated for 10 min at 30 °C. Meanwhile, bound receptors were washed 2× in supplied kinase buffer and then incubated with activated CaMKII phosphorylation mix (+A/PMSF) for 45 min at 30 °C with gentle agitation. After incubation, the kinase suspension was removed, and resins were washed 2× in wash buffer. Bound receptors were then eluted in 40 μL 1× LSB prepared in ultrapure water.

### 2.10. Protein Gels

All IP samples were separated on Bio-Rad Mini-Protean TGX precast gels (Bio-Rad, Hercules, CA, USA) using established protocols. Separated protein eluates were then stained using Simply Blue Safe Stain (Invitrogen). Samples were run alongside a protein molecular weight marker, and bands of the appropriate molecular weights were excised from the gel, transferred to microfuge tubes, and stored at −20 °C until processing for proteomics. Approximate molecular weights for the nAChR subunits and variants are as follows: α4-nAChR ~75 kDa (HEK transients and from mouse brain), β2-nAChR-YFP ~75 kDa, α4-nAChR-YFP ~100 kDa, and β2-nAChR ~55 kDa (HEK transients and from mouse brain). All processing was done in a clean environment and ultrapure water was used to prepare all buffers and reagents.

### 2.11. Protein Digestion for LC-MS/MS

Gel bands were first cut into small pieces and subjected to the following washes with agitation: 50% (*v*/*v*) acetonitrile (5 min), 50% (*v*/*v*) acetonitrile/10 mM NH_4_HCO_3_ (30 min). Gel pieces were dried with a speed vacuum, resuspended in 30 µL of 10 mM NH_4_HCO_3_/0.2 µg digestion grade trypsin (Promega), and incubated for 16 h at 37 °C. Peptides were acidified with 0.1% (*v*/*v*) trifluoroacetic acid (TFA) prior to mass spectrometry analysis.

### 2.12. Protein Identification by LC-MS/MS

Reverse phase liquid chromatography tandem mass spectrometry (RP-LC-MS/MS) was performed using a NanoACQUITY (Waters Corporation, Milford, MA) ultra-performance liquid chromatography (UPLC) coupled to a Q Exactive Plus Hybrid Quadrupole-Orbitrap (ThermoFisher Scientific, San Jose, CA, USA) mass spectrometer. Peptides were loaded onto a nanoACQUITY (Waters Corporation, Milford, MA, USA) UPLC Symmetry C18 trapping column (180 µm × 20 mm) at a flowrate of 5 µL/min prior to separation on a nanoACQUITY (Waters Corporation, Milford, MA, USA) Peptide BEH C18 column (75 µm × 250 mm). Mobile phase A and B compositions were 0.1% (*v*/*v*) formic acid in water and 0.1% (*v*/*v*) formic acid in acetonitrile, respectively. Peptides were eluted over 120 min with a mobile phase B gradient (6–20%) at a column temperature of 37 °C and a flow rate of 300 nL/min. Precursor mass scans (300 to 1500 *m*/*z* range, target value: 3 × 10^6^, maximum ion injection times: 45 ms) were acquired and followed by HCD-based fragmentation (normalized collision energy: 28). A resolution of 70,000 at *m*/*z* 200 was used for MS1 scans, and up to 20 dynamically chosen, most abundant precursor ions were fragmented (isolation window: 1.7 *m*/*z*). The tandem MS/MS scans were acquired at a resolution of 17,500 at *m*/*z* 200 (target value: 1 × 10^5^, maximum ion injection times: 100 ms). Mass spectrometry raw spectra were searched against the Mascot algorithm (Matrix Science, London, UK) using Proteome Discoverer software (v 2.2.0.388, ThermoFisher Scientific, San Jose, CA, USA). The search criteria were the following: precursor mass tolerance, 10.0 ppm; fragment mass tolerance, 0.020 Da; enzyme, trypsin; maximum missed cleavage sites, 2; variable modifications, carbamidomethyl (C), oxidation (M), phosphorylation (STY), propionamide (C).

### 2.13. Quantitative Data Analysis

Searched data was imported into Scaffold (v 4.8.7, Proteome Software Inc., Portland, OR, USA) software for validation of peptide and protein identifications. Peptide and protein identifications were accepted above a 95% and 99% probability threshold, respectively. Proteins containing less than two peptides per protein were filtered out, and proteins sharing redundant peptides were grouped. Peptide and protein probabilities were calculated by the Scaffold Local FDR algorithm and Protein Prophet algorithm [26], respectively. For label-free quantitative analysis, the Scaffold Q+ (v 4.8.7, Proteome Software Inc., Portland, OR, USA) function was used. Median-normalization of precursor ion intensities was performed across samples, which were then log-transformed and weighted by an adaptive intensity weighting algorithm. After removal of peptides not meeting the threshold criteria the following number of spectra were used for label-free quantitation: Experiment 1 (66%), 77,423 (quantitative)/117,463 (total); Experiment 2 (69%), 108,557 (quantitative)/158,253 (total); Experiment 3 (57%), 114,821 (quantitative)/200,002 (total). Analysis settings were specified for the following categories: Analysis type, Intensity-based; Experiment type, Between subjects (Independent Groups). Quantitation Preferences were selected as follows: (1) Minimum Value Preference; Use Minimum Absolute Intensity, false; Minimum Absolute Intensity, 0.0; Minimum Value: 0.01; (2) Condenser Preferences; Use Intensity Weighting, true; Use Standard Deviation Estimation, true; Use Non-Exclusive Peptides, true; (3) View Preference; View Type, Log_2_ Ratio; (4) Normalization Preference; Calculation Type, Median; Blocking Level, Unique Peptides; Use Inter Experiment Normalization, true; Use Intra Sample Normalization, false; Use Peptide Normalization, false; Use Protein Average As Reference, true; Use Iterative Normalization, true; Spectrum Quality Filter, no filter. For annotation of protein PostTranslational Modification (PTM) sites, Scaffold PTM (v 4.8.7, Proteome Software Inc., Portland, OR, USA), which integrated the MS/MS results exported from Scaffold/Scaffold Q+. For differential phosphorylation analysis, phosphorylated peptide spectral counts were first normalized to the total spectral counts for each protein. This value was then normalized to the total spectral counts for the entire sample.

### 2.14. Statistical Analysis

For all replicates, normalized quantitative values for the “treated” group were compared to those of the “control” group. Statistical analyses were performed in GraphPad Prism v7.01 (La Jolla, CA, USA) using two-tailed Student *t*-tests. The level for significance was set at *p* < 0.05.

## 3. Results

### 3.1. Co-Expression of nAChR Subunits and CaMKII in HEK Cells

**Figure 1:**
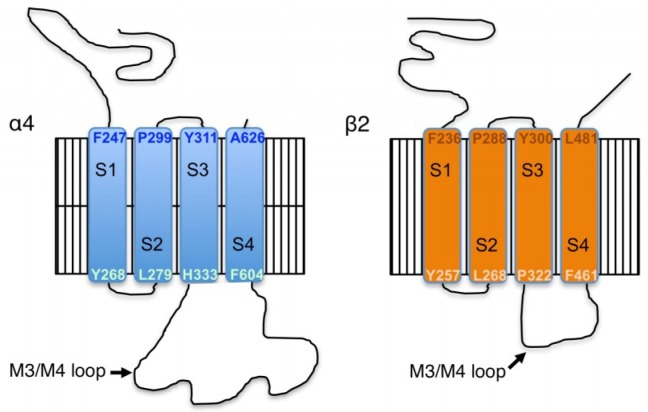
Amino acid structure of mouse α4/β2 nAChR subunits. Membrane topology of the mouse α4/β2 nAChR shows the boundaries of the intracellular domains. The intracellular M3/M4 loop of the α4 subunit is the longest of all the nAChR subtypes, and is the site of most identified protein–protein interactions [8,16].

Trafficking and activity of high-affinity nAChRs can be regulated by phosphorylation [7,14,15]. The intracellular loop between the 3rd and 4th transmembrane domain of the α4 subunit (M3/M4 loop) is the longest found among all nAChR subunits and has been identified as a locus for protein–protein interactions and phosphorylation (Figure 1). Further, activation of nAChRs can increase intracellular calcium levels in the cells on which they are expressed, and α4/β2 nAChRs are physically associated with several isoforms of the calcium-dependent kinase CaMKII in mouse and human brain [6,8]. In order to determine whether the α4 or β2 nAChR subunits can be phosphorylated by CaMKII, we co-expressed untagged and YFP-tagged nAChR subunits in HEK cells (2 independent replicates per condition), with or without CaMKIIα-mRuby, and used mass spectrometry to identify phosphorylation sites on the nAChR. To control for any effects of the fluorescent tag, parallel experiments were performed using α4-YFP with untagged β2 and untagged α4 with β2-YFP. Results did not differ depending on which subunit was fluorescently tagged, so data on phosphorylation sites were pooled between the two studies for statistical evaluation.

**Figure 2:**
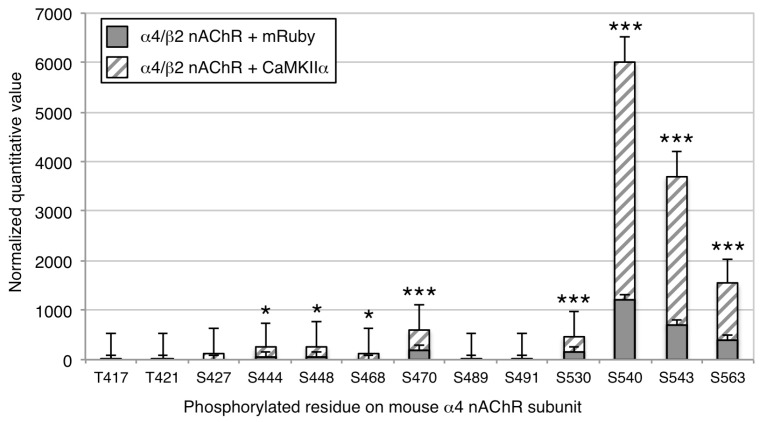
Co-expression of α4/β2 nAChRs and CaMKII in HEK cells. Phosphorylated residues on tagged α4 and β2 nAChR subunits co-expressed with mRuby or CaMKIIα -mRuby in HEK cells were identified by mass spectrometry following immunoprecipitation and separation by gel electrophoresis. Phosphorylation level was normalized to total subunit protein. Phosphorylation of 5 serine residues on the α4 subunit (S470, S530, S540, S543, S563) could be identified in HEK cells co-expressing mRuby, and 8 serine residues showed a significant increase in phosphorylation in HEK cells co-expressing CaMKIIα (S444, S448, S468, S470, S530, S540, S543, S563). No phosphorylation of the β2 subunit was detected. * *p* < 0.05; *** *p* < 0.005. Error bars represent standard error of the mean; *n* = 6/condition.

Following transfection, nAChRs were immunoprecipitated and subunits were separated by gel electrophoresis. Gels were Coomassie-stained, bands of the appropriate size for the untagged and tagged α4 and β2 nAChR subunits were excised, and proteins were digested and subjected to mass spectrometry. We identified 8 serine residues on the α4 subunit with significantly increased phosphorylation following co-expression with CaMKIIα-mRuby, compared to those co-expressing mRuby alone (Figure 2, see Supplementary Materials for representative spectra). Phosphorylation of the β2 subunit on S445 was identified in the baseline condition in HEK cells, but there was no significant phosphorylation of this residue in the CaMKIIα-mRuby condition. The phosphorylated residues identified in the α4 subunit were all in the intracellular M3/M4 loop of the protein (see Figure 1) and include serine 444 (S444), S448, S468, S470, S530, S540, S543, and S563 (Figure 2, Table 1), as was the S445 phosphorylation site in the β2 subunit (Table 1).

**Figure 3:**
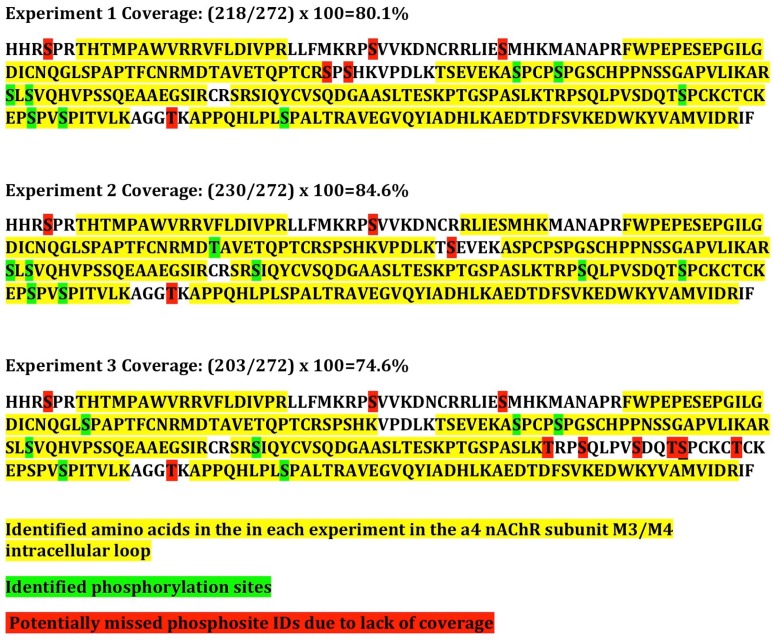
Coverage of the α4 nAChR subunit M3/M4 intracellular loop across experiments. Coverage of the large intracellular loop of the α4 subunit is diagrammed for each experiment for direct comparison. Yellow: identified amino acids; Green: identified phosphorylation sites; Red: serine or threonine residue that was not covered and might represent a missed phosphorylation site.

The coverage of the α4 subunit intracellular M3/M4 loop was ~80%, suggesting that the majority of physiologically relevant sites of phosphorylation were likely identified, and only 6 serine or threonine residues in the intracellular loop were uncovered (Figure 3). However, overall coverage of the α4 subunit was 61% and of the β2 subunit was 43%, so additional sites of phosphorylation could be present, but not identified in this experiment.

### 3.2. CaMKII and PKA Can Phosphorylate nAChRs In Vitro

**Figure 4:**
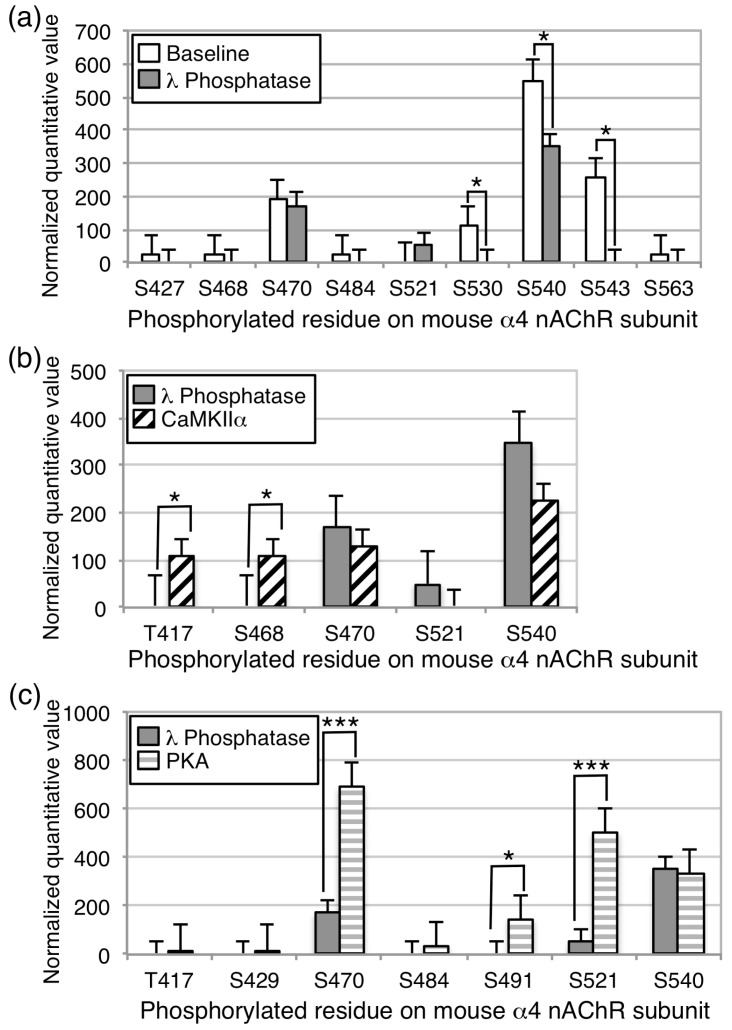
In vitro phosphorylation of α4/β2 nAChRs by CaMKII or PKA. The α4 and β2 nAChR subunits were co-expressed in HEK cells, isolated by immunoprecipitation, and subjected to mass spectrometry. Phosphorylation level was normalized to total subunit protein. (**a**) At baseline, there was a high level of phosphorylation of S470, S530, and S540 on the α4 subunit, and incubation with lambda phosphatase dephosphorylated S540 and S543 to undetectable levels. (**b**) Incubation with CaMKIIα in the presence of calcium and calmodulin increased phosphorylation of T417 and S468 on the α4 subunit significantly. (**c**) Incubation with PKA in the presence of cyclic AMP increased phosphorylation of S470, S491, and S521 significantly. * *p* < 0.05; *** *p* < 0.005. Error bars represent standard error of the mean; *n* = 6/condition.

Since multiple kinases are found in all cell types, phosphorylation of the nAChR subunits may have resulted directly through CaMKII activity, or indirectly through activation of other kinases expressed in HEK cells. We, therefore, performed an in vitro phosphorylation experiment using α4/β2 nAChRs immunoprecipitated from HEK cells following dephosphorylation using lambda phosphatase. Following immunoprecipitation and dephosphorylation, tagged or untagged nAChRs were incubated with CaMKIIα in the presence of calcium and calmodulin or PKA. nAChRs were then separated by gel electrophoresis, and bands were excised for evaluation by mass spectrometry. As above, parallel experiments were performed using α4-YFP with untagged β2 and untagged α4 with β2-YFP, and no differences were found, so data for tagged and untagged subunits were pooled. At baseline, four highly phosphorylated serine residues were identified on the α4 subunit, S470, S530, S540, S543 (Figure 4, see Supplementary Materials for representative spectra). Except for S470, phosphorylation of these sites was decreased or nearly eliminated following incubation with lambda phosphatase (Figure 4a). Incubation with CaMKIIα resulted in phosphorylation of T417 and S468 above the phosphatase condition, whereas phosphorylation of S470 and S540 were detected, but were not higher than the phosphatase condition (Figure 4b, Table 1). Incubation with PKA resulted in phosphorylation of S470, S491, and S521 above the phosphatase condition (Figure 4c, Table 1). Of these sites, S468, S470, and S540 were detected when nAChR subunits and CaMKII were co-transfected into HEK cells as described above (Figure 2). The coverage of the α4 subunit intracellular M3/M4 loop was ~85%, suggesting that the majority of physiologically relevant phosphorylation sites were likely identified, but 6 serine or threonine residues in the intracellular loop were uncovered in this experiment (Figure 3). No significant changes in phosphorylation were identified on the β2 subunit, although phosphorylation of T375 was detected in both the CaMKII and PKA conditions, and not at baseline (Table 1). Coverage of the α4 and β2 subunits was 55% and 38%, respectively, so additional sites of phosphorylation could be present, but not identified in this experiment. These findings identify distinct phosphorylation sites on the α4 subunit for PKA and CaMKIIα, as well as sites that may be phosphorylated at baseline by these or other kinases.

### 3.3. Phosphorylation of nAChRs In Vivo

**Figure 5:**
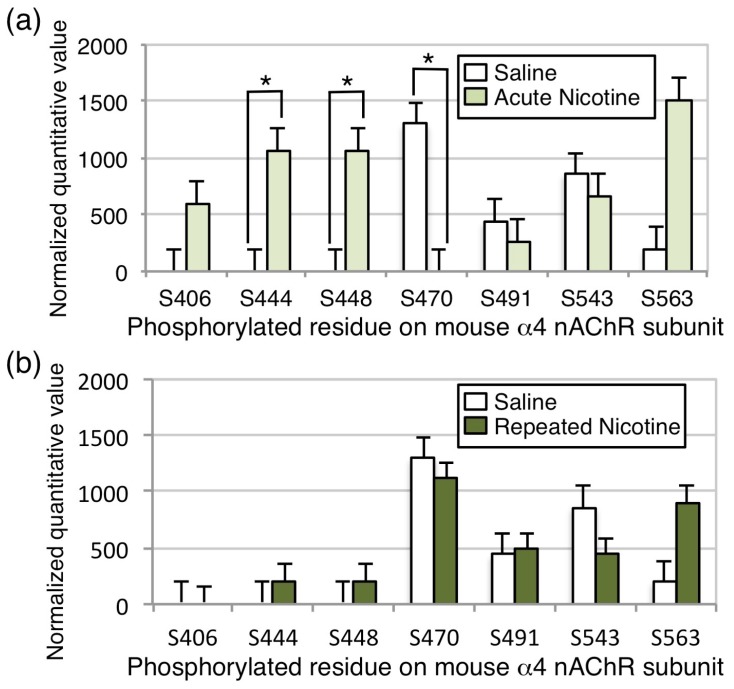
Phosphorylation of α4/β2 nAChRs in vivo following nicotine exposure. nAChRs were immunoprecipitated from mouse brain homogenates using a monoclonal antibody raised against the α4 subunit, isolated by gel electrophoresis, and bands corresponding to the α4 and β2 subunits were excised and subjected to mass spectrometry. Phosphorylation level was normalized to total subunit protein. Phosphorylation of S491, S543, and S563 on the α4 subunit was detected in brain homogenates from saline treated mice. (**a**) Following acute nicotine exposure in vivo, phosphorylation of S444 and S448 was significantly increased, whereas phosphorylation of S470 was significantly decreased to undetectable levels. (**b**) Following chronic exposure to nicotine, no significant differences from baseline phosphorylation were observed in the α4 subunit. No phosphorylation of the β2 subunit was detected. * *p* < 0.05. Error bars represent standard error of the mean; *n* = 10/condition.

We next determined whether the phosphorylation sites identified as CaMKII or PKA targets in vitro were also phosphorylated in vivo under conditions in which nAChRs could be activated by nicotine. Using monoclonal antibodies raised against the α4 subunit, we immunoprecipitated native α4/β2* nAChRs from mouse brain following saline administration, a single nicotine dose in a novel environment (0.5 mg/kg), or chronic nicotine in the drinking water, a regimen known to increase locomotor activity in a dopamine-dependent manner [23]. In mice that had been handled and placed in a novel environment following saline administration, we once again identified phosphorylation of S470, S491, and S543 in the α4 subunit (Figure 5, see Supplementary Materials for representative spectra). Acute nicotine administration resulted in a significant increase in phosphorylation of S444 and S448, whereas repeated nicotine exposure resulted in no significant differences over baseline, but detectable phosphorylation of S444, S448, S470, S491, S543, and S563 (Figure 5). Increases in S406 and S563 were observed, but did not reach significance. Coverage of the M3/M4 loop of the α4 subunit was ~75% in this experiment, but 10 serine or threonine residues in the intracellular loop were uncovered in this experiment, including S521 and S530 which were identified as a potential PKA site and in HEK cells, respectively (Figure 3). Interestingly, S470 in the α4 subunit was significantly phosphorylated following saline administration, and phosphorylation was reduced to undetectable levels following acute nicotine administration, then returned to baseline levels following repeated nicotine exposure. No sites of phosphorylation were identified on the β2 subunit. Coverage of the α4 subunit was 53%, and of the β2 subunit was 47%, so additional sites of phosphorylation could be present, but not identified in this experiment.

## 4. Discussion

**Figure 6:**
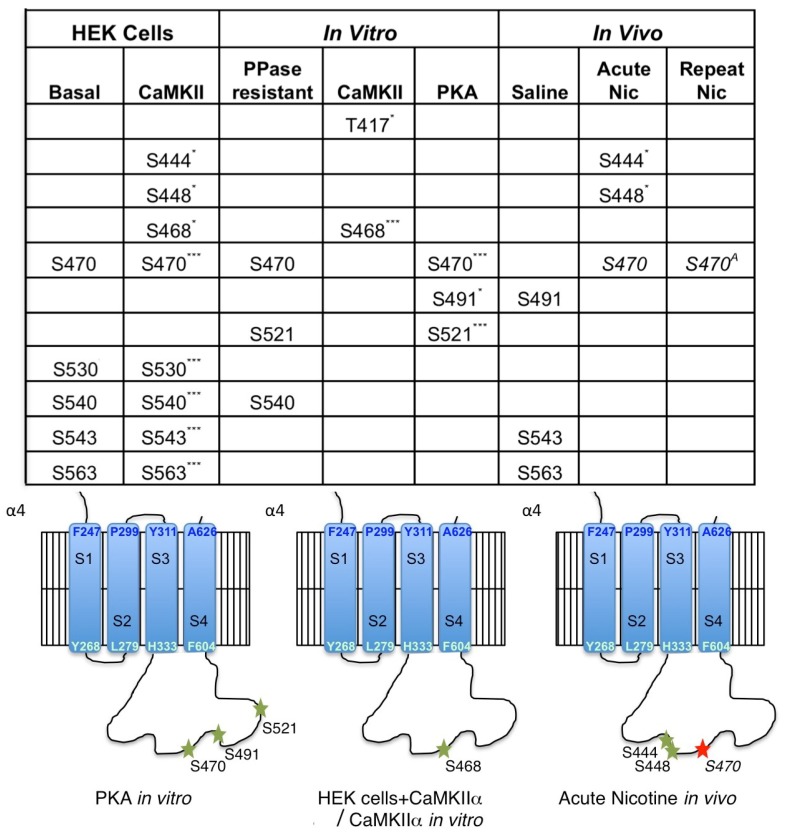
Summary of α4 nAChR subunit phosphorylation sites. Comparison of the sites detected in the α4 subunit across experiments and topological site of phosphorylation sites identified in vitro and in vivo. Green: sites with increased phosphorylation; Red/italics: site with decreased phosphorylation; ^A^: no difference from baseline; * *p* < 0.05; *** *p* < 0.005.

These experiments identify previously described and novel sites on the mouse α4 nAChR subunit, and the first report of specific residues on the β2 nAChR subunit, that can be phosphorylated in cells after heterologous expression with CaMKIIα, or by CaMKIIα or PKA in vitro (summarized in Figure 6). In addition, we report the first identification of in vivo nAChR phosphorylation at baseline and in response to nicotine exposure in mouse brain (Figure 5). Despite phosphorylation of the mouse β2 nAChR subunit on S445 in HEK cells and T375 in vitro by CaMKIIα or PKA, no phosphorylation in vivo at baseline or following nicotine exposure was detected, although coverage of the subunit was not complete, so additional sites may not have been revealed.Co-transfection of the α4 and β2 nAChR subunits with CaMKIIα on the α4 subunit in HEK cells induced significant phosphorylation of 8 sites on the mouse α4 subunit, all of which are conserved in the human α4 nAChR subunit and 3 of which (S444, S448 and S468) have not been reported previously. Of these, 2 sites (S470 and S540) match the minimal requirements for phosphorylation by CaMKII (RXXS/T, where R is arginine and T is threonine; [27]), however, incubation of isolated α4/β2 nAChR with CaMKIIα in vitro did not result in significant phosphorylation of these residues. Instead, in vitro CaMKIIα phosphorylated one site identified in the HEK cell experiment (S468) and a novel site not identified previously (T417). T417 conforms to the minimal consensus sequence for CaMKII phosphorylation, but S468 has a different basic residue (K (lysine) rather than R) at the −3 position.

The in vitro experiment identified two additional residues that were phosphorylated by PKA (S491 and S521) that have not been described as substrates for this kinase previously, along with highly significant phosphorylation of S470. A number of studies have identified S470 as an important site of phosphorylation on the α4 nAChR subunit [7,11,12,28,29,30]. These studies show that S470 can be phosphorylated by both PKA and PKC in vitro and after co-transfection in cultured cells. Our in vitro experiments confirm that S470 is a substrate for PKA, and suggest it is not a direct substrate for CaMKIIα, although activation of CaMKII appears to lead to increased phosphorylation of this residue in HEK cells, possibly through indirect activation of another kinase or decreased activity of a phosphatase. Phosphorylation of S470 on the α4 subunit by PKA is necessary for recruitment of the scaffolding protein 14-3-3, and this association increases stability of the α4/β2 nAChR and contributes to upregulation following nicotine exposure [7]. In addition, phosphorylation of the α4 subunit by PKC increases activity of the α4/β2 nAChR by enhancing recovery from desensitization following agonist exposure [28,31]. We observed baseline phosphorylation of S470 on the α4 nAChR subunit in cells and in mouse brain across experiments, however, acute nicotine exposure in vivo significantly decreased the phosphorylation of S470, whereas chronic exposure returned the phosphorylation state to baseline levels in the mouse brain. Thus, the decreased phosphorylation of S470 following acute nicotine exposure, observed here, is likely to result in decreased activity of the receptor, whereas the recovery to baseline following chronic exposure could be important for nAChR upregulation, which is observed in mouse brain following the chronic nicotine exposure regimen used here [23,32]. Note that, in all experiments, phosphorylation level was normalized to total subunit protein.

The decrease in phosphorylation of the S470 site on the mouse α4 subunit following acute nicotine treatment in vivo suggests that stimulation of nAChRs may result in activation of a protein phosphatase that dephosphorylates this residue. This observation is consistent with experiments showing that nicotine acting through nAChRs can activate the calcium-dependent phosphatase calcineurin in cultured cortical neurons [33]. In addition, activity of calcineurin is required for nicotine-induced locomotor sensitization in rats [34], suggesting that this decrease in nAChR phosphorylation could be behaviorally relevant.

The consistent association between α4/β2 nAChRs and several CaMKII isoforms in mouse and human brain prompted us to investigate whether these receptors were a substrate for phosphorylation by this kinase. Neither of the phosphorylation sites identified following in vitro phosphorylation of α4/β2 nAChRs with CaMKIIα were identified in vivo at baseline or following nicotine exposure, suggesting that the nAChR may not be a major substrate for CaMKII in the mouse brain at baseline or under that conditions of nicotine exposure tested here. However, S444, S448, and S563 were phosphorylated both when CaMKIIα was co-expressed with the α4 and β2 nAChR subunits in HEK cells and in mouse brain. The physical interaction between α4/β2 nAChRs and CaMKII could result in activation of the kinase and phosphorylation of other protein targets. Therefore, the increased phosphorylation of α4/β2 nAChRs, when co-transfected with CaMKIIα in HEK cells, could be the result of a protein kinase cascade that indirectly results in phosphorylation of the nAChR by PKA and other unidentified kinases. Alternatively, the association between α4/β2 nAChRs and CaMKII may be important for other cell biological functions, such as localization of the receptor to particular intracellular compartments or the plasma membrane. Interaction with scaffolding proteins, such as 14-3-3, contributes to trafficking of nAChRs in a PKA-dependent manner [7], and CaMKII can serve as a binding protein to target other proteins to specific intracellular membranous compartments, such as synaptic vesicles [35]. Thus, α4/β2 nAChRs may be regulated by association with CaMKII in mouse brain, even if they are not an efficient substrate for phosphorylation by the enzyme.

## 5. Conclusions

In summary, this phosphoproteomic study has identified novel phosphorylation sites on the mouse α4 nAChR and β2 subunits, and is the first instance of identification of a subset of α4 nAChR residues phosphorylated, in vivo, in mouse brain tissue. Further, acute nicotine exposure increases the phosphorylation of two residues on the α4 subunit (S444 and S448), but decreases phosphorylation of a very well-characterized residue (S470) that contributes to surface trafficking and resistance to desensitization of α4/β2 nAChRs in cultured neurons [7,10,11,12]. Taken together, these studies suggest that nAChR activity initiates intracellular signaling cascades that can alter receptor activity. Furthermore, although it is not yet clear whether α4/β2 nAChRs are a substrate for CaMKIIα, which interacts physically with the receptor, these results suggest that CaMKIIα can affect phosphorylation of the α4 subunit indirectly in cells. Future studies using purified enzymes in vitro, co-transfection studies in cells, or specific stimuli in vivo will be necessary to identify the kinases that phosphorylate these novel sites. The functional consequences of these phosphorylation events on receptor assembly, trafficking, and function should also be evaluated in cells and in vivo.

## Figures and Tables

**Table 1:** Sequence of phosphorylation sites identified in the mouse α4 and β2 nAChR subunits.

Site	Sequence ^1^	Observed Previously ^2^	Conserved in Human	Predicted CaMKII Site ^3^
T417	…RMDTAVE…	No	No	Yes
S444	…EKASP…	No	Yes (S441)	No
S448	…PSPG…	No	Yes (S445)	No
S468	…KARSLSVQH…	No	Yes (S464)	No
S470	…KARSLSVQH…	Yes	Yes (S467)	Yes
S491	…RSRSIQ…	Yes	Yes (S488)	Yes
S521	…TRPSQLP…	No	No	No
S530	…DQTSPC…	Yes	Yes (S527)	No
S540	…KEPSPVSP…	Yes	Yes (S538)	Yes
S543	…KEPSPVSP…	Yes	Yes (S541)	No
S563	…LPLSPAL…	Yes	Yes (S561)	No

^1^ Phosphorylated residue is underlined. ^2^ Sites of phosphorylation in the α4 nAChR subunit identified in **7, 11, 12, 28**–**30**. ^3^ CaMKII sites predicted using the Phyre2 site: http://www.sbg.bio.ic.ac.uk/phyre2/.

## Supplementary Materials

The following are available online at http://www.mdpi.com/2227-7382/6/4/42/s1.
