# Supplementary material for: Evaluation of the Phosphoproteome of Mouse Alpha 4/Beta 2-Containing Nicotinic Acetylcholine Receptors In Vitro and In Vivo
Source: Proteomes. 2018 Oct 15;6(4):42. doi: 10.3390/proteomes6040042 (PMC6313896; doi:10.3390/proteomes6040042)
Supplement: Supplementary file 1 [file proteomes-06-00042-s001.pdf]

Supplementary Legend

Representative spectra for T417, S444, S448, S468, S470, S491, S521, S530, S540, S543 and S563. Red text highlights the b-ions and blue text highlights the y-ions.

T417

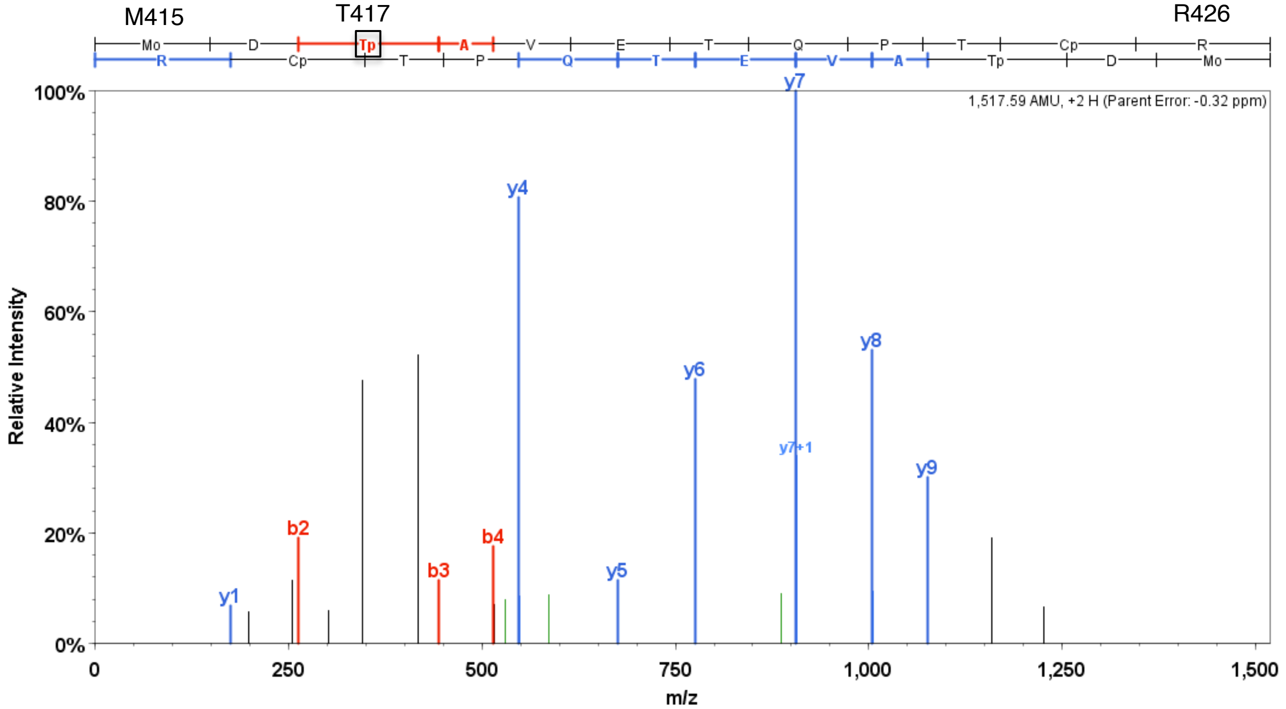

S444 & S448

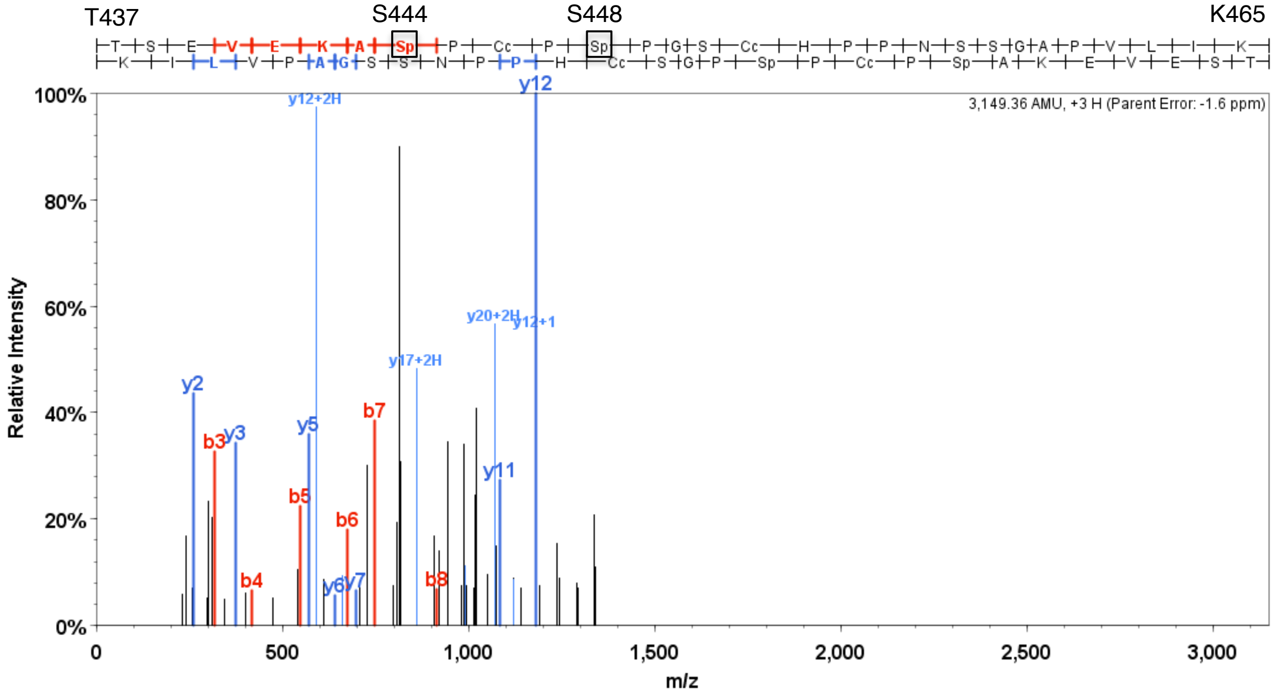

S468

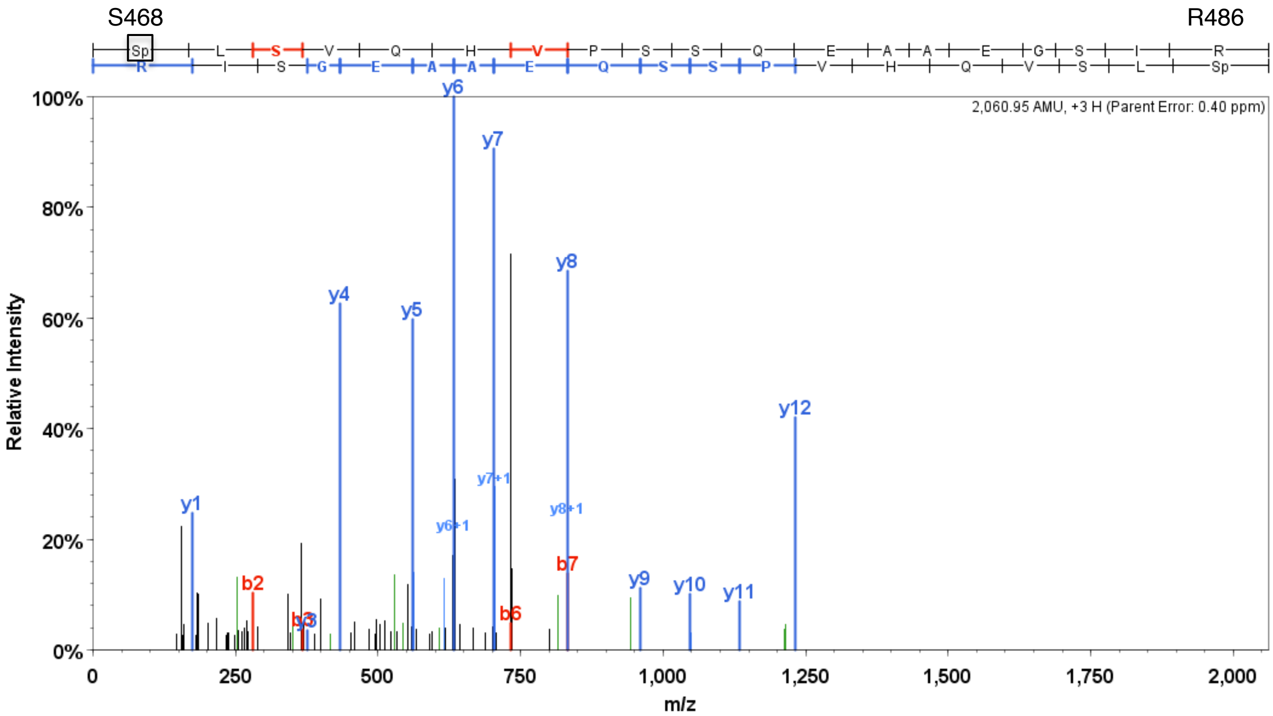

S468
R486
Sp L S V Q H V P S S Q E A A E G S I R
R I S G E A A E Q S S P V H Q V S L Sp
2,060.95 AMU, +3 H (Parent Error: 0.40 ppm)
Relative Intensity
m/z
y1 b2 b3 y4 y5 y6 y6+1 y7 y7+1 b6 b7 y8 y8+1 y9 y10 y11 y12

S470

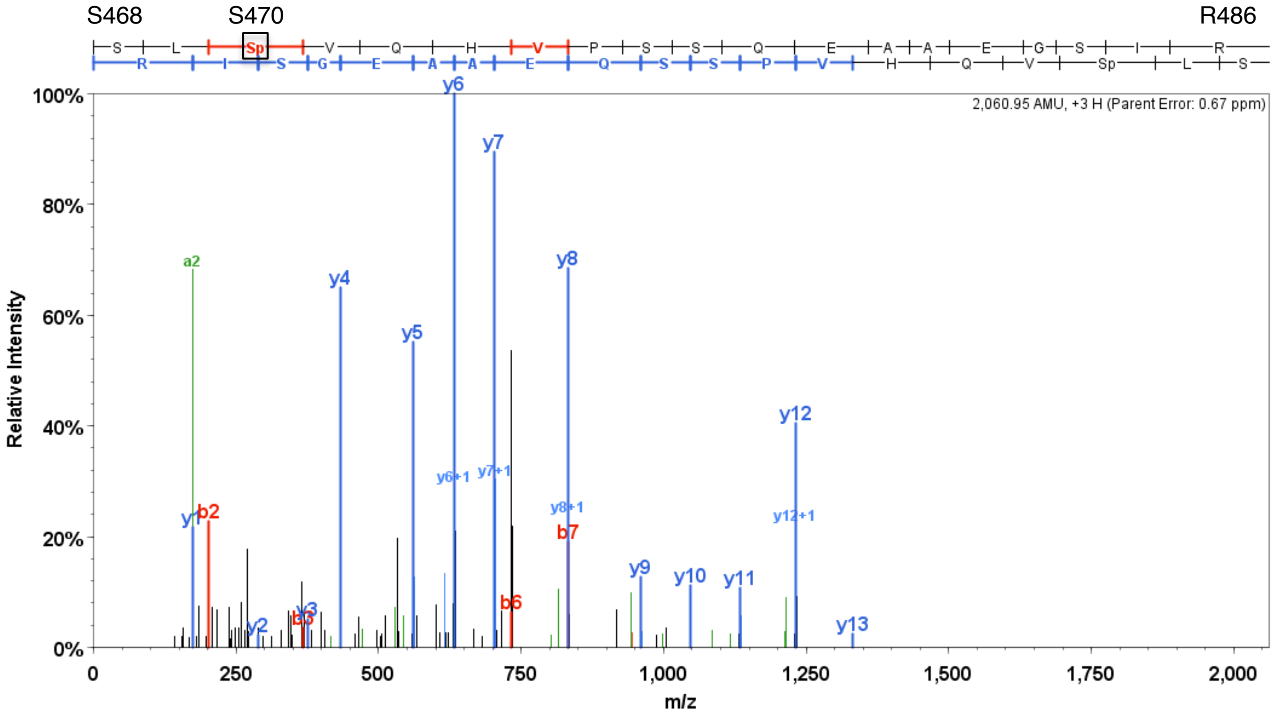

S468
S470
R486
S L Sp V Q H V P S S Q E A A E G S I R
R I S G E A A E Q S S P V H Q V Sp L S
2,060.95 AMU, +3 H (Parent Error: 0.67 ppm)
Relative Intensity
m/z
a2 y1 b2 y2 y3 b3 y4 y5 y6 y6+1 y7 y7+1 b6 b7 y8 y8+1 y9 y10 y11 y12 y12+1 y13

S491

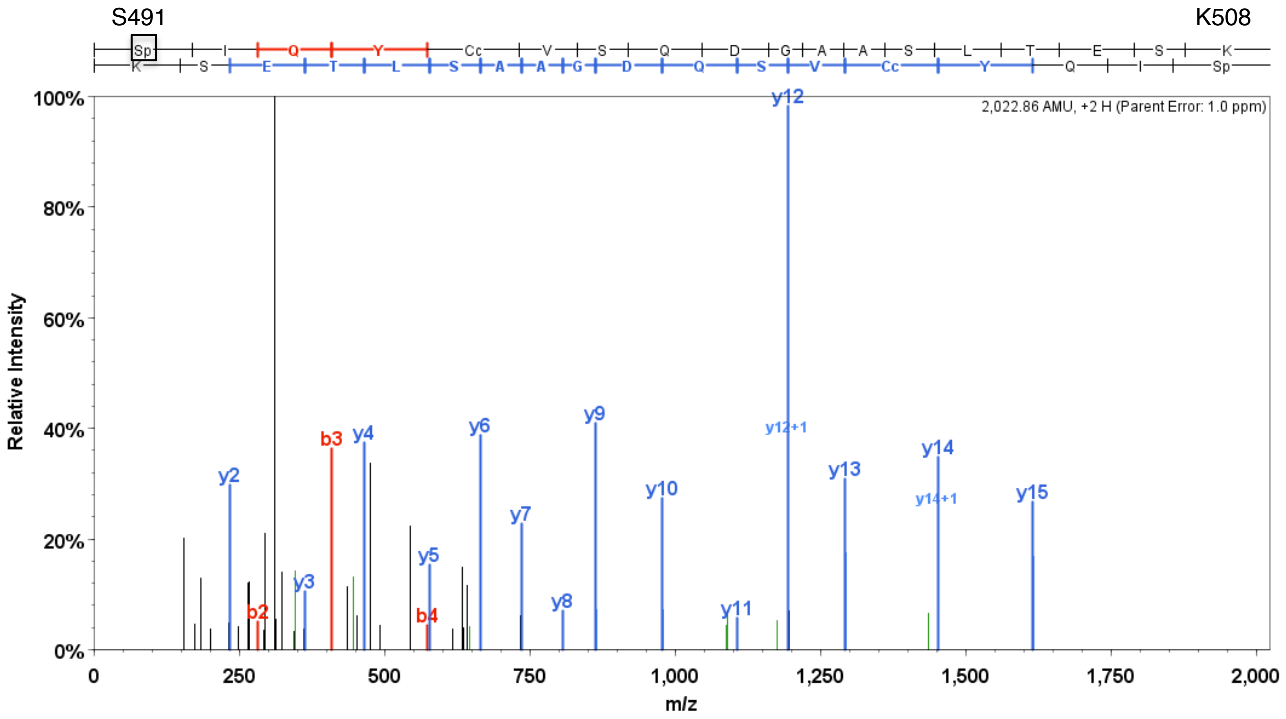
S491
K508
2,022.86 AMU, +2 H (Parent Error: 1.0 ppm)
Relative Intensity
m/z

S521

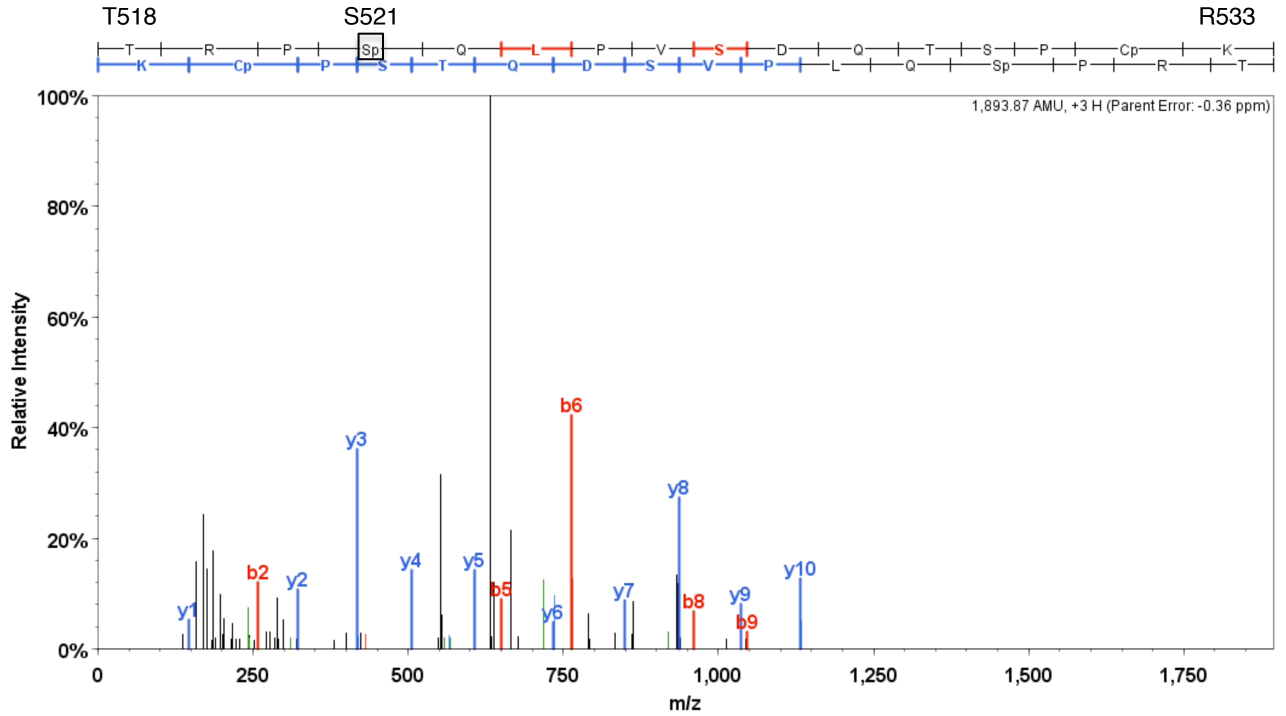
T518
S521
R533
1,893.87 AMU, +3 H (Parent Error: -0.36 ppm)
Relative Intensity
m/z

S530

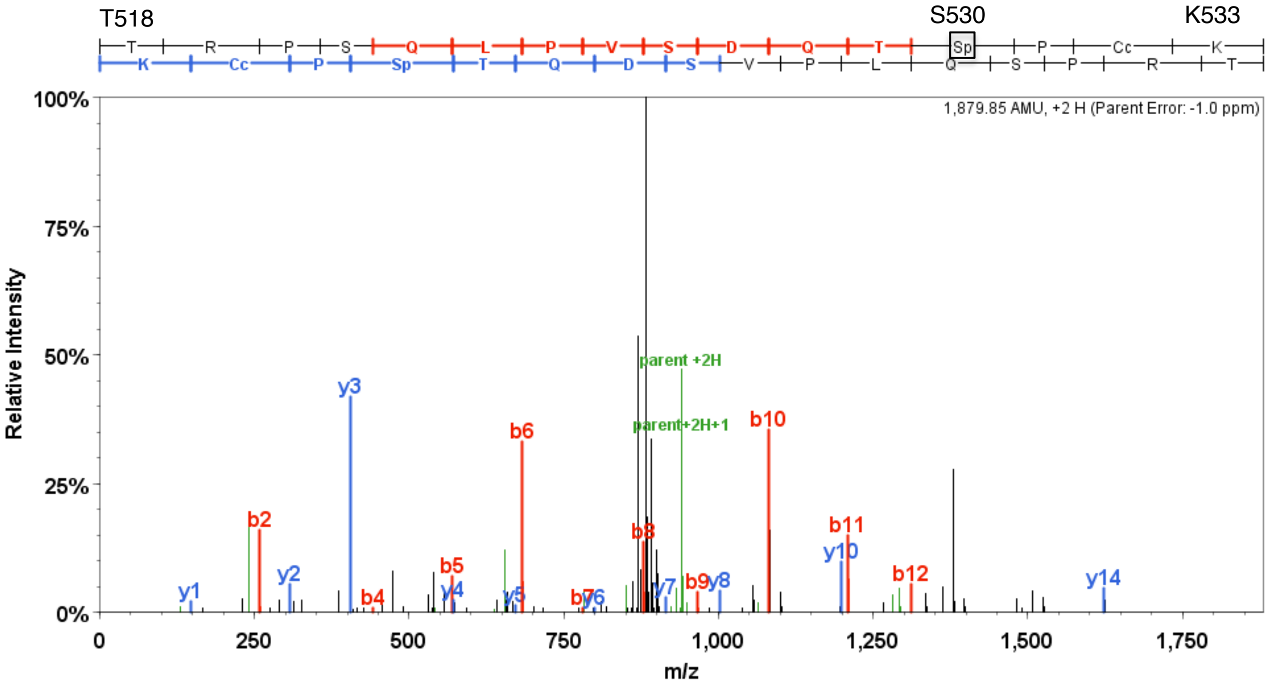
T518
S530
K533
1,879.85 AMU, +2 H (Parent Error: -1.0 ppm)
Relative Intensity
m/z
parent +2H
parent+2H+1
y1
y2
y3
y4
y5
y6
y7
y8
y10
y14
b2
b4
b5
b6
b7
b8
b9
b10
b11
b12

S540

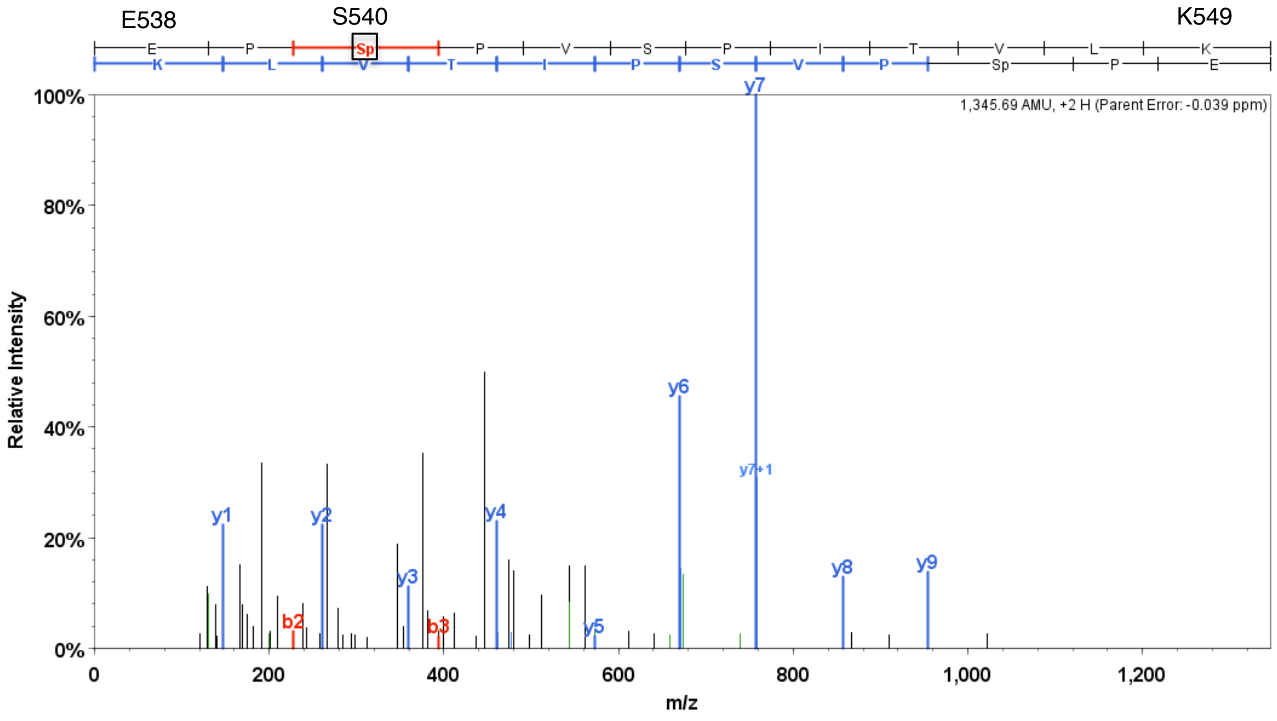
E538
S540
K549
1,345.69 AMU, +2 H (Parent Error: -0.039 ppm)
Relative Intensity
m/z
y1
y2
y3
y4
y5
y6
y7
y7+1
y8
y9
b2
b3

S543

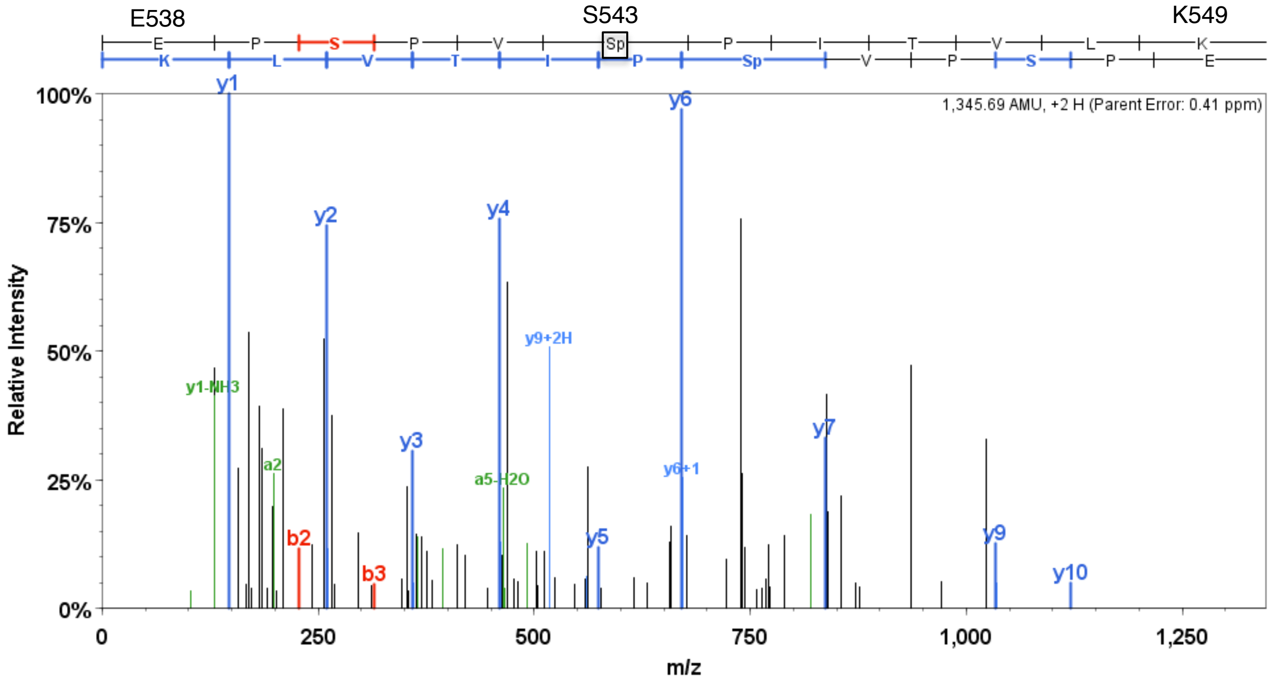
E538
S543
K549
E P S P V Sp P I T V L K
K L V T I P Sp V P S P E
1,345.69 AMU, +2 H (Parent Error: 0.41 ppm)
100%
75%
50%
25%
0%
Relative Intensity
0
250
500
750
1,000
1,250
m/z
y1
y6
y2
y4
y9+2H
y1-NH3
y3
a2
a5-H2O
y6+1
y7
b2
y5
y9
b3
y10

S563

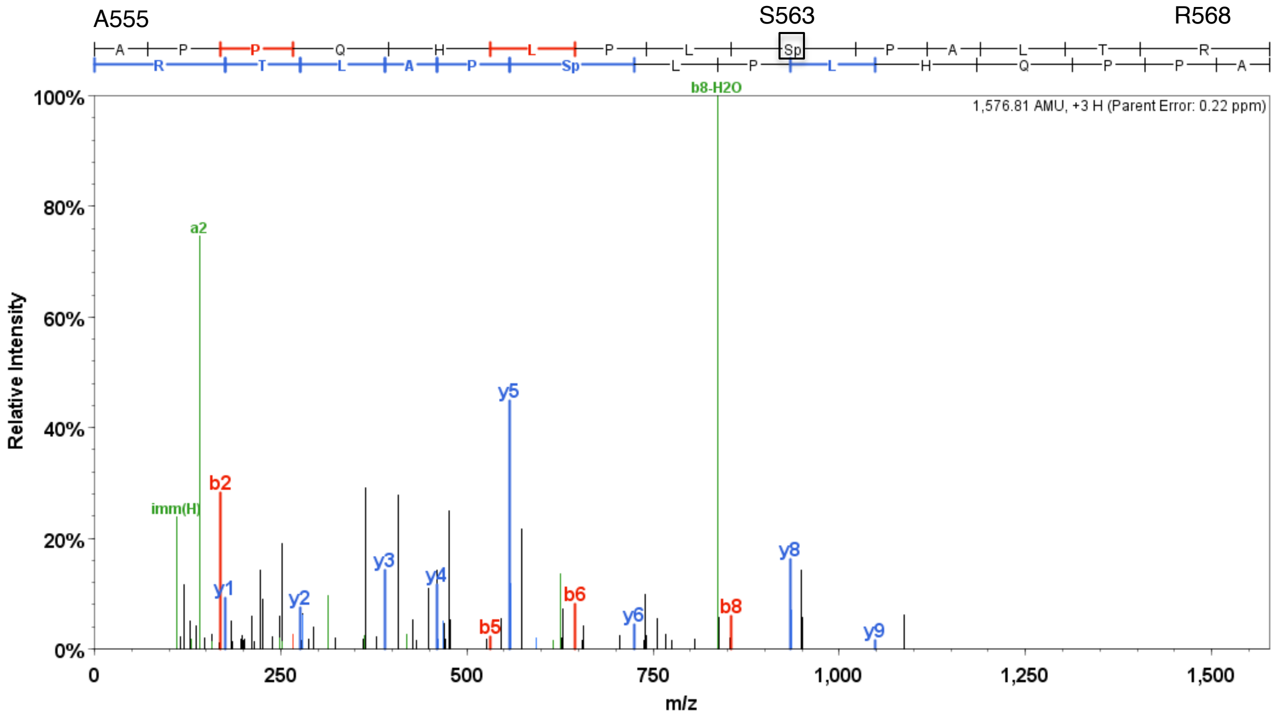
A555
S563
R568
A P P Q H L P L Sp P A L T R
R T L A P Sp L P L H Q P P A
1,576.81 AMU, +3 H (Parent Error: 0.22 ppm)
100%
80%
60%
40%
20%
0%
Relative Intensity
0
250
500
750
1,000
1,250
1,500
m/z
b8-H2O
a2
y5
b2
imm(H)
y3
y4
y1
y2
y8
b6
b8
y6
b5
y9
